# Minimal clinically important difference for the 6-min walk test: literature review and application to Morquio A syndrome

**DOI:** 10.1186/s13023-017-0633-1

**Published:** 2017-04-26

**Authors:** Rudolf Schrover, Kathryn Evans, Roberto Giugliani, Ian Noble, Kaustuv Bhattacharya

**Affiliations:** 1 SYNEVi Pty Limited, Level 4, Suite 402, 15 Help Street, Chatswood, NSW 2067 Australia; 2 BioMarin Pharmaceutical Australia Pty Ltd, Sydney, NSW Australia; 3 Medical Genetics Service/HCPA, Department of Genetics/UFRGS and INAGEMP, Porto Alegre, Brazil; 4 Noble Pharma Consulting, Sydney, NSW Australia; 5 Genetic Metabolic Disorders Service, Sydney Childrens’ Hospital Network, Westmead, NSW Australia; 6 Discipline of Child and Adolescent Health, Sydney University, Sydney, NSW Australia

**Keywords:** Enzyme replacement therapy, Morquio A syndrome, Mucopolysaccharidosis IVA, Endurance, Six-minute walk test, Minimal clinically important difference, MPS IVA

## Abstract

Morquio A syndrome is an ultra-rare, inherited lysosomal storage disorder associated with progressive, multi-systemic clinical impairments, causing gradual loss of functional capacity and endurance, impaired quality of life, and early mortality. Studies in Morquio A patients have used the 6-min walk test (6MWT) to assess functionality and endurance and to evaluate disease progression or efficacy of treatment. The objective of the present study was to review minimal clinically important differences (MCIDs) for the 6MWT reported for disease states that widely use the 6MWT to evaluate clinical benefit and to discuss the results in view of the challenges in estimating MCID for ultra-rare diseases, using the case of elosulfase alfa in Morquio A patients. A systematic literature search was performed using Embase and Medline to identify studies specifically estimating the MCID using either anchor-based or distribution-based methods. A total of 19 publications on 17 studies were identified; none of these included patients with Morquio A syndrome or the wider disease category of lysosomal storage disorders. Therefore, the MCIDs determined by studies in patients with respiratory, cardiovascular, or musculoskeletal disease were compared to changes in the 6MWT seen in Morquio A patients in the pivotal phase 3 clinical trial of elosulfase alfa enzyme replacement therapy. The literature review showed a mean MCID for the 6MWT of 7% change (range 3–15%) in studies using anchor-based methods and a 9% change (range 4–16%) using distribution-based methods. Results of the elosulfase alfa clinical trial and its extension showed a placebo-adjusted 14.9% improvement in the 6MWT from baseline at week 24, which was greater than the mean MCID based on the results of the systematic literature review. After 2 years, 6MWT distance increased by a mean of 20.7% from baseline in a modified per-protocol population, versus a reduction of 6.9% in comparable untreated patients from the MorCAP natural history study over the same period. Although further research is required to establish the MCID of the 6MWT in Morquio A patients, the presented data provide further evidence for the positive effect of elosulfase alfa in this patient population.

**Electronic supplementary material:**

The online version of this article (doi:10.1186/s13023-017-0633-1) contains supplementary material, which is available to authorized users.

## Background

Morquio A syndrome, also known as mucopolysaccharidosis (MPS) IVA, is an ultra-rare, severely debilitating and progressively life-threatening inherited disorder (ORPHA309297) [1]. It is caused by mutations of the gene *N*-acetylgalactosamine-6-sulfatase (GALNS), resulting in impaired degradation of the glycosaminoglycans (GAGs) keratan sulfate (KS) and chondroitin-6-sulfate (C6S). As a result, KS and C6S accumulate in the lysosomes of cells throughout the body, which leads to widespread cellular, tissue, and organ dysfunction [1–4].

All patients with Morquio A syndrome suffer from a range of serious and debilitating morbidities across multiple domains including skeletal and joint abnormalities, short stature, impairments in respiratory function (restrictive and obstructive lung disease), cardiac function (valvular regurgitation and stenosis, low stroke volume), hearing, and vision, abdominal manifestations (hepatomegaly, hernias), and dental abnormalities, whilst retaining normal intelligence [1, 5–9]. The significant limitations in respiratory function, cardiac function, and musculoskeletal compromise result in low endurance, debilitating fatigue, pain, poor quality of life, and early mortality [4, 5, 10]. Due to low endurance and functional capacity limitations, many patients with Morquio A syndrome end up using a wheelchair or walking aid [1]. Historically, survival in patients with Morquio A syndrome has been severely reduced with the majority of patients dying in their second or third decade of life, mainly due to cardiorespiratory failure [4]. Less than 5% of patients live beyond 40 years [5].

Impaired endurance and functional capacity, both of which are important patient-relevant outcomes for Morquio A patients, may be measured clinically through the six-minute walk test (6MWT), which is a simple, commonly used, standardized measure of endurance [11]. It evaluates the global and integrated responses of all body systems involved during exercise, including the pulmonary and cardiovascular systems, systemic and peripheral circulation, and neuromuscular function. The American Thoracic Society (ATS) Consensus Statement describes the validated approach for performing the 6MWT [11].

**Fig. 1:**
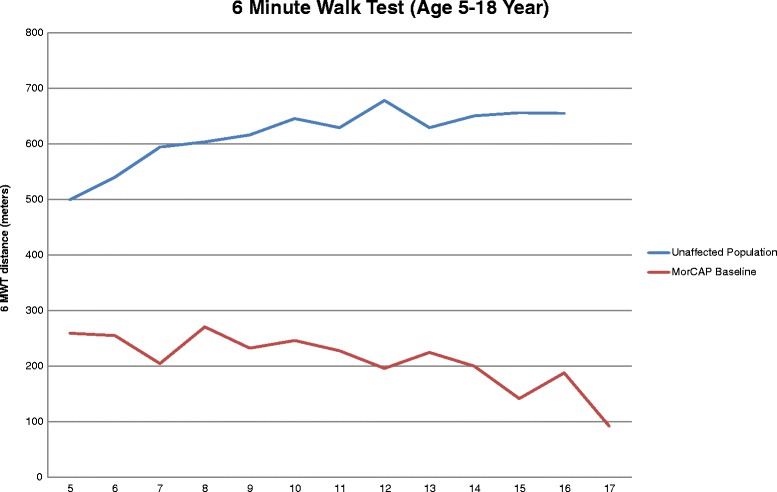
6MWT distance in untreated Morquio A patients and age-matched healthy controls. Source: [4, 14]

Baseline data from the Morquio A Clinical Assessment Program (MorCAP) study, a longitudinal natural history study examining the outcomes of patients with Morquio A, has shown that endurance and functional capacity, as measured by the 6MWT, are severely impaired in patients with Morquio A syndrome [4]. The overall mean (± SD) 6MWT distance of 212 ± 152 m in MorCAP was significantly lower than that of healthy individuals aged 4–16 years, reported as between 470 and 664 m [12, 13]. Moreover, a cross-sectional analysis showed that the mean 6MWT distance generally decreases with increasing age in patients with Morquio A syndrome: mean 6MWT distance was 251.6 m ± 121.5 m in 0–4 year olds (*n* = 37), 232.5 m ± 140.1 m in 5–11 year olds (*n* = 127), 181.2 m ± 177.3 m in 12–18 year olds (*n* = 84), and 193.1 m ± 148.5 m in 18 year olds (*n* = 68), thus illustrating the progressive nature of the disease [4]. This progressive decline in 6MWT is further illustrated in Fig. 1, comparing untreated Morquio A subjects with the normal population [4, 14]. Further evidence that untreated Morquio A syndrome is progressive and characterized by a gradual decline in endurance has been provided by 1- and 2-year data from MorCAP, in which a general decline in 6MWT distance from baseline was observed over the course of the 2-year longitudinal study [15]. At each visit, mean 6MWT distances were found to generally decrease with increasing age with an annualized estimate of change (SE) in 6MWT from baseline of −4.86 ± 3.25 m across all subjects, and −6.84 ± 5.38 m for subjects meeting the inclusion/exclusion criteria of the Phase 3 clinical trial of elosulfase alfa (≥5 years of age with baseline 6MWT distance ≥30 and ≤325 m) [15]. For these reasons, the 6MWT was the primary endpoint in the pivotal Phase 3 randomized, double-blind, placebo-controlled MOR-004 clinical trial of elosulfase alfa enzyme replacement therapy (ERT). In this study, a statistically significant improvement of 22.5 m (95% CI 4.0, 40.9; *p* = 0.017) in 6MWT distance over placebo was demonstrated with elosulfase alfa 2.0 mg/kg/week at 24 weeks [10], which was sustained over 120 weeks [16]. This incremental change in the 6MWT represents a 14.9% placebo-adjusted change from baseline at 24 weeks (Additional file 1). At 2 years in the open-label extension trial MOR-005, 6MWT distance increased by a mean of 11.7% (SD 57.2) versus baseline in the Intent-to-treat (ITT) population and a mean of 20.7% (SD 57.0) versus baseline in the Modified-Per-Protocol (MPP) population (excluding patients who had orthopedic surgery during the extension study or missed ≥20% of their scheduled elosulfase alfa infusions), versus decreases of 7.2% (SD 35.7) and 6.9% (SD 32.7) over the same period in comparable untreated patients from the MorCAP natural history study (Additional file 2). In addition to the effects on 6MWT, long-term (2 years) treatment with elosulfase alfa was associated with a sustained reduction of around 60% in urinary KS, a mean increase of 6.8 stairs/min in the 3-min stair climb test (3MSCT), improvements in respiratory function (+9.2% for forced vital capacity, 8.8% for forced expiratory volume in 1 s, and 6.1% for maximum voluntary ventilation), and improvements in the patients’ abilities to perform activities of daily living (MPP population) [16–18].

A recent study by Lampe et al. [19] showed an association between 6MWT and quality of life in patients with Morquio A syndrome. The authors reported that an increase of 100 m in 6MWT distance was associated with a 0.2 increase in the quality of life utility score of the EuroQoL 5 domains, 5 levels (EQ-5D-5 L) questionnaire. There are currently no studies reporting the relationship between the 6MWT and survival in patients with Morquio A syndrome. However, many studies in widely varying diseases show a positive correlation between the 6MWT and survival, especially in diseases associated with compromised respiratory and cardiovascular function [20–26]. As Morquio A syndrome is associated with multi-systemic clinical impairment and the most common causes of death are due to cardiorespiratory failure, it is logical to assume from a biological perspective that the relationship between the 6MWT and survival is also present in patients with Morquio A syndrome.

Although an improvement or decline in the 6MWT distance seems important as such, the clinical relevance and impact on patients’ daily functioning of changes in the 6MWT distance observed in clinical trials remains to be established for Morquio A. A standard way of measuring clinical relevance is determining the minimal clinically important difference (MCID), which is the smallest difference that a patient would perceive as beneficial [27]. The objective of this study was to review published studies which aimed to estimate the MCID for the 6MWT in a variety of disease states that widely use the 6MWT to evaluate clinical benefit and to discuss the results in view of the challenges of estimating MCID in ultra-rare diseases using the case of elosulfase alfa in Morquio A patients.

## Methods

### A systematic review of the literature

Literature searches were conducted in April 2014 using Embase with data covering both Embase and Medline. The search terms used included all variations of ‘six-minute walk test’ (e.g., ‘walking test’; ‘6 min walk test’, or ‘6MWT’) and all forms of the term ‘minimal clinically important difference’ including ‘MCID’; ‘clinically important’, or ‘clinically significant’. The search was not restricted to Morquio A syndrome in order to identify studies in other diseases that could help interpret the clinical relevance of 6MWT as an outcome measure. Furthermore, the search was also not limited by study type or language. The retrieved articles were reviewed to identify studies that reported a threshold for the 6MWT that was considered clinically meaningful. The reference lists of all retrieved papers were reviewed for any additional studies that did not appear in the original literature search. The results for each of the identified studies were extracted and grouped according to the MCID estimation method used.

There are three general approaches for estimating the MCID: anchor-based, distribution-based, and consensus-based methods. These approaches measure a quantifiable change in the 6MWT. Anchor-based methods compare the change in a clinical measure to the change in a patient-related anchor such as a patient-reported outcome (PRO), typically a global assessment rating in which the patients rate themselves as “better”, “unchanged”, or “worse” [28]. Some studies have used death, hospitalization, or other clinical measures (e.g., FVC) as “anchors” [29–32]. Within the anchor-based approaches, four variations exist including the within- and between-patients score change as well as sensitivity and specificity approaches and the social comparison approach [28]. Distribution-based methods compare the change in an outcome to a standardized measure of variability such as the standard error of measurement (SEM), standard deviation (SD), effect size, or minimum detectable change (MDC). Full details on variations of the anchor- and distribution-based methods are described in a review by Copay A G, et al. [33]. Consensus-based methods, also called Delphi-based methods, use expert panel opinion to establish which numerical value constitutes the minimal clinically relevant change for an outcome measure [34].

## Results and discussion

**Fig. 2:**
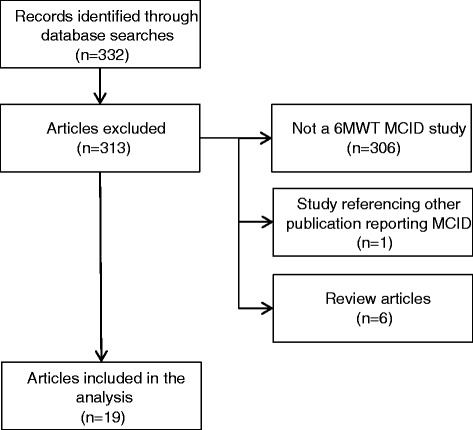
Flow chart summary of the systematic literature search. Studies that aimed to estimate the minimal clinically important difference (MCID) for the six-minute walk test (6MWT) in diseases relevant to Morquio A syndrome including respiratory, cardiovascular, and muscular diseases were included

A total of 19 publications on 17 studies which aimed to estimate the MCID for the 6MWT in diseases other than Morquio A syndrome were identified (Fig. 2). These comprised three broad categories, including respiratory disease (idiopathic pulmonary fibrosis [IPF], diffuse parenchymal lung disease, chronic obstructive pulmonary disease [COPD], non-cystic fibrosis bronchiectasis, emphysema), cardiovascular disease (chronic heart failure [CHF], coronary artery disease [CAD], pulmonary arterial hypertension [PAH]), and muscular disease (Duchenne muscular dystrophy [DMD]) (Table 1). One study was conducted in frail elderly Asian persons and was allocated to the category “Other”. Although no studies in Morquio A syndrome were identified, the disease categories are characteristic of the symptoms commonly experienced by patients with Morquio A syndrome. A total of 52 estimates of the 6MWT MCID were reported of which 40% (21/52) were determined using an anchor-based method approach while 52% (27/52) were estimated using a distribution-based method. In four cases (8%), the method to calculate MCID was not reported.

**Table 1:** Overview of studies that estimate the minimal clinically important difference (MCID) for the six-minute walk test (6MWT)

	Reference	Disease	*N*	Mean baseline 6MWT distance (m)	MCID determination method	
					Anchor-based	Distribution-based
1 & 2	du Bois, 2010 [29] & du Bois, 2011 [30]	IPF	822	392	x	x
3	Swigris, 2010 [31]	IPF	154^a^	373	x	x
4	Holland, 2009 [42]	Diffuse parenchymal lung disease	48^b^	403	x	x
5	Holland, 2010 [43]	COPD	75	359	x	x
6	Polkey, 2013 [32]	COPD	2112	369	x	
7	Puhan, 2008 [51]	COPD	460	361		x
8	Puhan, 2011 [44]	COPD	1218	348	x	x
9	Redelmeier, 1997 [45]	COPD	112	371	x	
10	Wise, 2005 [52]	Emphysema (COPD)	470	353		x
11	Lee, 2014 [46]	Non-cystic fibrosis bronchiectasis	37	551	x	x
12 & 13	Gremeaux, 2011 [48] and [47]	CAD	81	NA	x	x
14	Gilbert, 2009 [53]	PAH	207	339–347		x
15	Mathai, 2012 [49]	PAH	405	343	x	x
16	Henricson, 2013 [28]	DMD	24	370	x	
17	McDonald, 2013 [54]	DMD	174	358		x
18	Kwok, 2013 [50]	Frail elderly Asian persons	73	295	x	x
19	Taeger, 2013 [55]	CHF	NA	NA	NA	NA

*CAD* coronary artery disease, *CHF* chronic heart failure, *COPD* chronic obstructive pulmonary disease, *DMD* Duchenne muscular dystrophy, *IPF* idiopathic pulmonary fibrosis, *MCID* minimal clinically important difference, *NA* not available, *PAH* pulmonary arterial hypertension

^a^Only 123 patients had data at baseline and follow-up

^b^24 patients had IPF

### Absolute 6MWT MCID

**Fig. 3:**
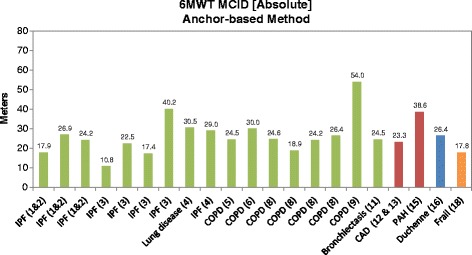
Absolute six-minute walk test (6MWT) minimal clinically important difference (MCID) using anchor-based methods. Sources: 1 & 2 = du Bois, 2010 [29] and du Bois, 2011 [30]; 3 = Swigris, 2010 [31]; 4 = Holland, 2009 [42]; 5 = Holland, 2010 [43]; 6 = Polkey, 2013 [32]; 8 = Puhan, 2011 [44]; 9 = Redelmeier, 1997 [45]; 11 = Lee, 2014 [46]; 12 & 13 = Gremeaux, 2011 [47] and Gremeaux, 2011 [48]; 15 = Mathai, 2012 [49]; 16 = Henricson, 2013 [28]; 18 = Kwok, 2013 [50]. The *green*, *red*, *blue*, and *orange* bars represent studies conducted in patients with respiratory, cardiovascular, muscular, and ‘other’ (i.e., frail elderly) diseases, respectively. CAD: coronary artery disease; COPD: chronic obstructive pulmonary disease; IPF: idiopathic pulmonary fibrosis; PAH: pulmonary arterial hypertension

The results of the studies estimating the MCID for the 6MWT using the anchor-based approach are presented in Fig. 3. The MCIDs as measured in absolute terms of meters walked ranged from 11–54 m across all studies. When considered separately by respiratory, cardiovascular, muscular, and other diseases, the MCID ranges were 11–54 m, 23–45 m, 26 m, and 18 m, respectively.

**Fig. 4:**
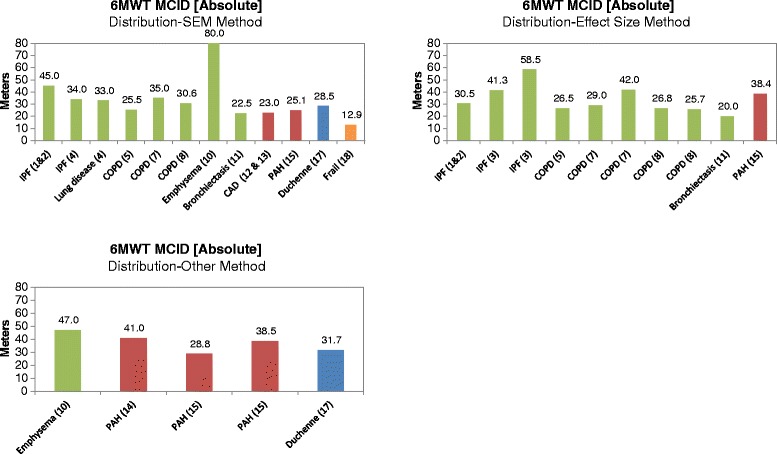
Absolute six-minute walk test (6MWT) minimal clinically important difference (MCID) using distribution-based methods. Sources: 1 & 2 = du Bois, 2010 [29] and du Bois, 2011 [30]; 3 = Swigris, 2010 [31]; 4 = Holland, 2009 [42]; 5 = Holland, 2010 [43]; 7 = Puhan, 2008 [51]; 8 = Puhan, 2011 [44]; 10 = Wise, 2005 [52]; 11 = Lee, 2014 [46]; 12 & 13 = Gremeaux, 2011 [47] and Gremeaux, 2011 [48]; 14 = Gilbert, 2009 [53]; 15 = Mathai, 2012 [49]; 17 = McDonald, 2013 [54]; 18 = Kwok, 2013 [50]. The *green*, *red*, *blue*, and *orange* bars represent studies conducted in patients with respiratory, cardiovascular, muscular, and other (i.e., frail elderly) diseases, respectively. CAD (coronary artery disease) COPD: chronic obstructive pulmonary disease; IPF: idiopathic pulmonary fibrosis; PAH: pulmonary arterial hypertension

The results of the studies estimating the MCID for the 6MWT using various distribution-based methods are presented in Fig. 4. Several different variations of the distribution-based methods were reported, including SEM, effect size, and ‘other methods’. The category ‘other methods’ are those studies that used variants of the standard deviation approach as well as those studies that did not clearly report the exact distribution-based method used. The range of MCIDs for the 6MWT using the SEM, effect size, and ‘other methods’ was similar to that seen in studies using the anchor-based approach with ranges between 13 and 45 m (excluding an 80 m outlier), 20–59 m, and 29–47 m, respectively. When the estimated MCIDs were considered by respiratory, cardiovascular, and ‘other’ diseases, the results were also similar. Although the absolute measures of the MCID for the 6MWT are informative, there are notable differences in terms of ability to walk between the patients enrolled in the studies considered in this analysis and those with Morquio A syndrome. The distance walked in the 6MWT at baseline in the MorCAP study was 212.6 ± 152.2 m [4], which is much lower than the average of 375 m for the clinical intervention studies included in this analysis. Note, however, that baseline 6MWT results were not reported for all included studies. Additionally, the influence of short stature on distance walked has not been estimated in any study, whereas the median Z-score for height for adults with Morquio A syndrome in the MorCAP study was −8.94 [4]. This severe short stature undoubtedly influences the total distance walked meaning that relative rather than absolute changes in distance walked are more indicative of therapy benefit compared with other studies.

In most studies, the absolute MCID was calculated without any specific intervention, so it can be considered as a measure of disease burden, with smaller 6MWT MCIDs representing a greater disease burden.

### Relative 6MWT MCID

In the absence of published studies reporting an MCID for Morquio A syndrome, it may be considered appropriate to view the MCID in relative change rather than absolute change from baseline to help interpret the clinical relevance of 6MWT changes. Therefore, the MCID was estimated as the required percentage change in meters from baseline for those studies reporting the results of the 6MWT at baseline. Since not all studies reported the results of the 6MWT at baseline, the number of studies included in the analysis was reduced to 14 studies reporting a total of 40 estimates of 6MWT MCID.

**Fig. 5:**
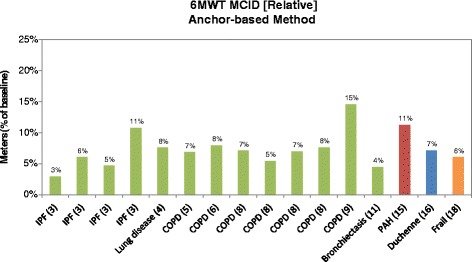
Relative six-minute walk test (6MWT) minimal clinically important difference (MCID) using Anchor-based methods. Sources: 3 = Swigris, 2010 [31]; 4 = Holland, 2009 [42]; 5 = Holland, 2010 [43]; 6 = Polkey, 2013 [32]; 8 = Puhan, 2011 [44]; 9 = Redelmeier, 1997 [45]; 11 = Lee, 2014 [46]; 15 = Mathai, 2012 [49]; 16 = Henricson, 2013 [28]; 18 = Kwok, 2013 [50]. The *green*, *red*, *blue*, and *orange* bars represent studies conducted in patients with respiratory, cardiovascular, muscular, and other (i.e., frail elderly) diseases, respectively. COPD: chronic obstructive pulmonary disease; IPF: idiopathic pulmonary fibrosis; PAH: pulmonary arterial hypertension

The results of the re-analyzed MCID studies using the anchor-based method are presented in Fig. 5. The MCID for the 6MWT as measured in relative terms is a mean of 7% (range 3–15%). When considered separately by respiratory, cardiovascular diseases, muscular disease, and ‘other’ the results were 3–15, 11, 7, and 6%, respectively.

**Fig. 6:**
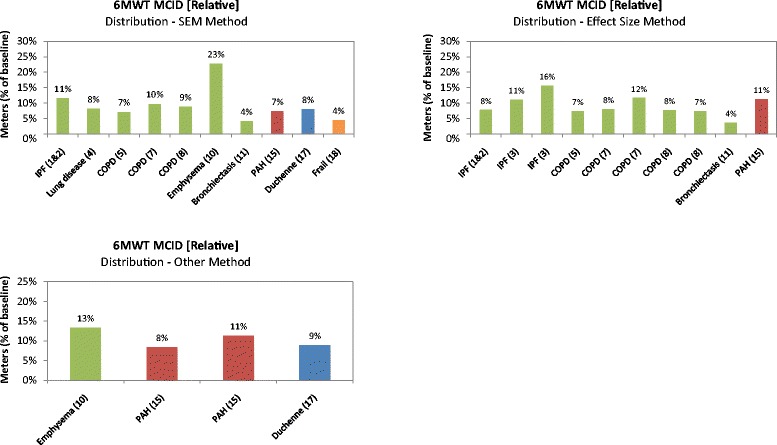
Relative six-minute walk test (6MWT) minimal clinically important difference (MCID) using distribution-based methods. Sources: 1 & 2 = du Bois, 2010 [29] and du Bois, 2011 [30]; 3 = Swigris, 2010 [31]; 4 = Holland, 2009 [44]; 5 = Holland, 2010 [43]; 7 = Puhan, 2008 [51]; 8 = Puhan, 2011 [44]; 10 = Wise, 2005 [52]; 11 = Lee, 2014 [46]; 15 = Mathai, 2012 [49]; 17 = McDonald, 2013 [54]; 18 = Kwok, 2013 [50]. The *green*, *red*, *blue*, and *orange* bars represent studies conducted in patients with respiratory, cardiovascular, muscular, and other (i.e., frail elderly) diseases, respectively. COPD: chronic obstructive pulmonary disease; IPF: idiopathic pulmonary fibrosis; PAH: pulmonary arterial hypertension

The relative re-analyzed MCID for the 6MWT estimated using the various distribution-based methods are presented in Fig. 6. The mean relative change using this method is 9% with a range of 4–16% (excluding a 23% outlier) across all diseases. When the relative MCIDs were considered by respiratory, cardiovascular, muscular, and ‘other’ diseases, the results were 4–16, 7–11, 8–9, and 4%, respectively.

### Applying MCIDs from other chronic diseases to Morquio A syndrome

The average relative MCID for the 6MWT in other chronic diseases is similar in anchor-based (mean 7%; range 3–15%) and distribution-based methods (mean 9%; range 4–16%). In the absence of any specific published studies on the MCID for the 6MWT in Morquio A syndrome, these values may serve as an evidence-based proxy until an MCID using 6MWT or other relevant clinical or patient-reported measures for Morquio A syndrome is established.

The mean 6MWT distances at baseline in the Phase 3 randomized, double-blind, placebo-controlled clinical trial of elosulfase alfa (MOR-004) were 203.9 and 211.9 m for patients treated with elosulfase alfa and placebo, respectively. At week 24, the estimated treatment effect compared with placebo was 22.5 m, corresponding with a change from baseline of 14.9%. The mean percentage improvement from baseline was 23.9% and 8.7% for patients treated with elosulfase alfa 2.0 mg/kg/week and placebo, respectively. In the long-term MOR-005 extension study, the mean change from baseline after 2 years in the 6MWT was 11.7% in the ITT and 20.7% in the MPP population, versus a reduction of 7.2% and 6.9%, respectively in comparable untreated patients from the MorCAP natural history study [16]. The observed treatment difference is greater than the 7–9% described above and greater than or at the upper limit of the range of MCIDs calculated for other diseases using anchor-based and distribution-based methods. Of note, one of the inclusion criteria of the MOR-004/005 trial was a baseline 6MWT distance ≥30 and ≤325 m [10]. However, not all Morquio A patients are ambulatory. The value of the 6MWT is not maintained in case of walking difficulties due to limb deformities, even when the patient has a relatively good muscular and cardiovascular state.

It is important to qualify the limitations of applying the MCID for the 6MWT of other chronic diseases to Morquio A syndrome. Specifically, patients with Morquio A syndrome experience respiratory, cardiovascular, and musculoskeletal impairment and as such their ability to perform the 6MWT is more severely compromised compared with those enrolled in studies investigating the MCID of the 6MWT in only one body system (e.g., respiratory, cardiovascular). While the above-mentioned range of relative MCIDs for other chronic diseases may provide an indication of the 6MWT MCID in Morquio A syndrome, it is likely that the true MCID for the 6MWT in patients with Morquio A syndrome is lower. As patients with Morquio A syndrome tend to have a lower functional capacity at baseline than the other diseases included in the present study, smaller improvements in 6MWT distance might be clinically important, as previously demonstrated for patients with DMD with varying baseline 6MWT results [35]. The analysis also does not take into account the age of patients included in the different studies, which range from young children of 4–12 years (DMD studies) to elderly patients of around 60–70 years of age for most respiratory diseases. This might, at least partly, explain the large differences in baseline 6MWT distances between studies. The age of the Morquio A patients included in the MOR-004/005 studies ranged from 5–57 (mean 14.4) years. In addition, the relevance of 6MWT MCIDs for the patients’ health-related quality of life (HRQoL) in the chronic diseases included in the present analysis is unclear [36]. The 6MWT is generally not used as a measure of HRQoL for these conditions. Although 6MWT distance has been found to be consistently associated with symptoms such as peak work capacity and physical activity in these conditions, correlations with HRQoL are generally weak [37]. For Morquio A, a sub-analysis of data from a PRO study (*N* = 24) recently showed a strong correlation between 6MWT distances and EuroQoL (EQ)-5D-5 L utility scores (*R* = 0.815; *P* < 0.0001) [19]. However, the association between changes in the 6MWT and HRQoL needs to be established. Of note, several pivotal placebo-controlled clinical trials in patients with lysosomal storage disorders similar to Morquio A (i.e., MPS I, MPS VI, and late-onset Pompe disease) have used the 6MWT, or the comparable 12MWT, as a primary endpoint to determine treatment efficacy for the ERT being assessed [38, 39]. Regulators and payers agreed that the 6MWT outcomes provided enough evidence for a clinically relevant treatment effect for these diseases leading to the approval and reimbursement of these medications for treating the designated disorders. The fact that the long-term improvements in the 6MWT in Morquio A patients in the MOR-004/005 study were accompanied by improvements in respiratory function and the ability of patients to perform activities of daily living also provides further evidence for the clinical relevance of 6MWT results in these patients [17, 18]. It remains to be elucidated whether the improvements in respiratory function contribute to the improvements in 6MWT outcomes. Other mechanisms, such as increases in muscle strength, joint movement, or pain may also be involved.

Further research is required to directly establish the MCID of the 6MWT in patients with Morquio A syndrome as a reliable measure of treatment outcome. However, this entails selection of the most appropriate method first, taking into account the limitations of currently used MCID determinations. As illustrated by the wide distribution in MCIDs between the studies included in the present analysis, often within the same diseases state, different analysis methods produce different MCIDs. Whereas anchor-based methods yield different MCIDs depending on the PRO scale used and the arbitrary selection of grouping of scale levels, MCIDs obtained with distribution-based methods depend on the measure of statistical variability used in the analysis [33]. Another limitation of anchor-based methods is that the PRO measures used to calculate MCIDs are subjective, often not validated for the disease, and subject to recall bias [33]. In addition, PRO changes generally depend on baseline level, with patients with greater level of disability showing more improvement. Major issues of distribution-based methods are that outcomes are sample-specific, i.e., depend on the variability of results in the sample studied, and do not address the question of clinical importance for the patient [33].

## Conclusions

The identification of clinically relevant outcomes is critical in the evaluation of the efficacy of therapeutic interventions. From a physician’s, but also a payer’s and regulator’s perspective, it is important to understand the medical consequences of a treatment and the limitations of the disease to properly assess clinical improvements. Ideally each disease should have its own MCID determined. However, establishing evidence of clinical effectiveness can be very challenging for ultra-rare diseases such as Morquio A syndrome due to the very small number of physicians with specialized expertise in the disease, limited understanding of the natural history, and difficulties in generating a large volume of evidence in randomized clinical trials [40]. In the absence of sufficient data, it can be valuable to compare outcomes in studies of ultra-orphan diseases with MCIDs for that outcome established for other, better characterized conditions (respiratory, cardiovascular, and muscular diseases). Applying this strategy to the 6MWT in Morquio A provided further evidence for the clinical relevance of elosulfase alfa ERT as a treatment option. The mean MCID for the 6MWT of 7–9% change identified in studies using anchor-based and distribution-based methods is considerably below the 14.9% improvement over placebo at week 24 in Morquio A patients treated with elosulfase alfa in the pivotal phase 3 study. This improvement was sustained with long-term treatment. However, as this approach is subject to several limitations, the next step is to directly establish the MCID of the 6MWT in patients with Morquio A syndrome. Considering the advantages and limitations of currently used MCID determinations [33], both anchor- and distribution-based methods should ideally be used. A PRO measure that has been used successfully in Morquio A patients, and might therefore be useful when establishing the MCID for the 6MWT in Morquio A patients using an anchor-based method, is the MPS Health Assessment Questionnaire (MPS-HAQ), an MPS-specific questionnaire that assesses the patient’s ability to perform activities of daily living [1, 41]. Other, generic, PRO measures that have been proven useful in these patients are the EQ-5D questionnaire (assessing mobility, self-care, usual activities, pain/discomfort, and anxiety/depression), and the Brief Pain Inventory Short Form (BPI-SF) and the Adolescent Pediatric Pain Tool (APPT) that assess pain intensity in adults and children/adolescents, respectively [1].

## Additional files

Additional file 1: Treatment effect estimates for ANCOVA of 6-min walk test (6MWT) percent change from baseline to week 24. Analysis population: Intent-To-Treat. (DOCX 12 kb)

Additional file 2: Percent change from MOR-004 baseline in 6-min walk test (6MWT) distance at 2 years in the MOR-005 Modified Per-Protocol (MPP) population (excluding patients who had orthopedic surgery during the extension study or missed ≥20% of their scheduled elosulfase alfa infusions) and comparable, untreated patients from the MorCAP natural history study. (DOCX 12 kb)

## Abbreviations

6MWT: 6-min walk test
APPT: Adolescent Pediatric Pain Tool
BPI-SF: Brief Pain Inventory Short Form (BPI-SF)
CAD: Coronary artery disease
CHF: Chronic heart failure
COPD: Chronic obstructive pulmonary disease
DMD: Duchenne muscular dystrophy
ERT: Enzyme replacement therapy
GAGs: Glycosaminoglycans
GALNS: N-acetylgalactosamine-6-sulfatase
IPF: Idiopathic pulmonary fibrosis
MCID: Minimal clinically important difference
MorCAP: Morquio A Clinical Assessment Program
MPS: Mucopolysaccharidosis
MPS-HAQ: MPS Health Assessment Questionnaire
NA: Not available
PAH: Pulmonary arterial hypertension
PRO: Patient-reported outcomes
