# Supplementary material for: Minimal clinically important difference for the 6-min walk test: literature review and application to Morquio A syndrome
Source: Orphanet J Rare Dis. 2017 Apr 26;12:78. doi: 10.1186/s13023-017-0633-1 (PMC5405472; doi:10.1186/s13023-017-0633-1)
Supplement: Supplementary file 1 — Treatment effect estimates for ANCOVA of 6-min walk test (6MWT) percent change from baseline to week 24. Analysis population: Intent-To-Treat. (DOCX 12 kb) [file 13023_2017_633_MOESM1_ESM.docx]

**Additional file 1:** Treatment effect estimates for ANCOVA of 6MWT percent change from baseline to week 24. Analysis population: Intent-To-Treat

|  |  | Elosulfase alfa 2.0 mg/kg/week  N=58 |
| --- | --- | --- |
| Week 24 change from baseline | LS mean change difference from placebo (SE) | 14.9 (6.20) |
|  | 95% CI for LS mean change difference | 2.7, 27.2 |
|  | p-value | 0.017 |

CI: confidence interval; LS: least square
