# Supplementary material for: Minimal clinically important difference for the 6-min walk test: literature review and application to Morquio A syndrome
Source: Orphanet J Rare Dis. 2017 Apr 26;12:78. doi: 10.1186/s13023-017-0633-1 (PMC5405472; doi:10.1186/s13023-017-0633-1)
Supplement: Supplementary file 2 — Percent change from MOR-004 baseline in 6-min walk test (6MWT) distance at 2 years in the MOR-005 Modified Per-Protocol (MPP) population (excluding patients who had orthopedic surgery during the extension study or missed ≥20% of their scheduled elosulfase alfa infusions) and comparable, untreated patients from the MorCAP natural history study. (DOCX 12 kb) [file 13023_2017_633_MOESM2_ESM.docx]

**Additional file 2:** Percent change from MOR-004 baseline in 6-minute walk test (6MWT) distance at 2 years in the MOR-005 Intent-to-treat (ITT) population and the Modified Per-Protocol (MPP) population (excluding patients who had orthopedic surgery during the extension study or missed ≥20% of their scheduled elosulfase alfa infusions) and comparable, untreated patients from the MorCAP natural history study

|  | ITT | |  | MPP | |
| --- | --- | --- | --- | --- | --- |
|  | MOR-005 | MorCAP |  | MOR-005 | MorCAP |
| Percent change at 2 years |  |  |  |  |  |
| N | 154 | 38 |  | 117 | 25 |
| Mean (SD) | 11.7 (57.2) | -7.2 (35.7) |  | 20.7 (57.0) | -6.9 (32.7) |
| Median (Q1, Q3) | 10.2 (-7.6, 33.4) | -2.6 (-19.4, 11.7) |  | 14.8 (-2.7, 36.5) | -6.9 (-18.7, 7.8) |
| Min, Max | -100.0, 268.8 | -100.0, 76.0 |  | -100.0, 268.8 | -100.0, 76.0 |
